# What is appropriate care? An integrative review of emerging themes in the literature

**DOI:** 10.1186/s12913-017-2357-2

**Published:** 2017-06-30

**Authors:** Joelle Robertson-Preidler, Nikola Biller-Andorno, Tricia J. Johnson

**Affiliations:** 1 Institute of Biomedical Ethics and History of Medicine, University of Zurich, Winterthurerstrasse 30, 8006 Zürich, Switzerland; 2 Department of Health Systems Management, Rush University, 1700 W. Van Buren Street, Suite 126B, Chicago, IL 60612 USA

**Keywords:** Appropriate care, Concept, Integrative review

## Abstract

**Background:**

Health care improvement efforts should be aligned in order to make a meaningful impact on health systems. Appropriate care delivery could be a unifying goal to help coordinate efforts to improve health outcomes and ensure system sustainability. A more complete understanding of how appropriate care is currently conceived in research and clinical practice could help inform a more integrated and holistic concept of appropriate care that could guide health care policy and delivery practices. We examined the current understanding of appropriate care by identifying its use and definitions in recently published literature.

**Methods:**

An integrated review of the practices, goals and perspectives of appropriate care in English language peer-reviewed articles published from 2011 to 2016. Inductive content analysis was used to describe emerging themes of appropriate care in articles meeting inclusion criteria.

**Results:**

This integrative review included empirical studies, reviews, and commentaries with various health care settings, cultural contexts, and perspectives. Conceptualizations of appropriate care varied, however most descriptions fell into five main categories: evidence-based care, clinical expertise, patient-centeredness, resource use, and equity. These categories were often used in combination, indicating an integrated understanding of appropriate care.

**Conclusions:**

An understanding of how appropriate care is conceptualized in research and policy can help inform an integrated approach to appropriate care delivery in policy and practice according to the relevant priorities and circumstances.

## Background

Rising health care costs and strained budgets underscore the need to ensure that scarce health care resources reach the people that most need them. Inappropriate care in the form of under-use, over-use, and misuse of health care services has been recognized by the Institute of Medicine as a barrier to health care quality [1] that plagues health care systems across the world [2–6] and ultimately reinforces health care disparities that lead to poor health outcomes. To help systems address these challenges, the Institute of Medicine (IOM) created a framework for health systems to bridge gaps in quality and improve outcomes by emphasizing the need for health systems to pursue care that is safe, effective, patient-centered, timely, efficient, and equitable [7]. Furthermore, the Institute for Healthcare Improvement (IHI) developed the Triple Aim of improving population health and patient experience of care while decreasing per capita costs to guide system improvement efforts [8]. Industrialized countries have sought to improve health care delivery through a variety of policies. For example, the Affordable Care Act in the United States seeks to expand access through mandatory health insurance and promote new models of care, such as Accountable Care Organizations, that foster cost-efficient and high quality care [9], though, such efforts are new and the results have been mixed [10]. In other countries, cost-effectiveness criteria for service coverage and pay-for-performance models (e.g. NHS England’s Quality Outcomes Framework for primary care [11]) have attempted to facilitate appropriate care delivery. Understanding how appropriate care delivery is understood and currently used in policy and research could help guide policy makers to take a comprehensive approach to delivering care that aligns with system, clinical, and patient perceptions of appropriate care and improves patient outcomes and experiences while curbing health care spending.

Appropriateness is a recognized element of health care system performance [12–14]. The World Health Organization defines appropriateness from a system’s perspective as care that is effective, efficient and in line with ethical principles of fair allocation [15]. Researchers and policy makers have made efforts to conceptualize and measure appropriate care, both prospectively through the development of evidence-based guidelines [16–18] and retrospectively by assessing guideline adherence for specific conditions [18–20]. A scoping literature review by Sanmartin and colleagues (2008) found that the concept of appropriate care has been chiefly operationalized as the net clinical benefit to the average patient using the RAND/UCLA Appropriateness Method, however, definitions and application of appropriateness varied by setting and service [21].

Although appropriate care has been recognized as an important element of high quality care delivery, the concept remains a patchwork concept with no uniform scope or meaning [21]. In addition, the patient perspective and considerations of patient preferences and values have been largely neglected [21]. A more integrative view of appropriate care delivery could help systems to create effective policies to support clinical practices that can more effectively meet patients’ needs.

The purpose of this paper is to provide a contemporary snapshot of how appropriate care is understood in the post-US health reform world by identifying major themes of appropriate care that can help frame a more comprehensive approach to improving health system performance.

## Methods

We conducted an integrative review of recently peer-reviewed literature that focused on appropriate care delivery. Data was coded and analyzed using inductive content analysis to identify major categories to describe how appropriate care is used and conceptualized in research and practice.

### Literature search method

We searched Scopus, PubMed, and Medline/Ovid for English-language articles published from 2011 to 2016. Although appropriate care is a dynamic and evolving concept based on government, policy, and market forces [22], the objective of this review was to identify how appropriate care is currently understood and is limited to papers published in the six years following the enactment of the Appropriate Care Act. Search terms included “appropriate care,” “appropriateness of care,” and “care appropriateness.” Because there were no correlating MeSH terms, we searched author keywords which have been found to have correlation with MeSH terms [23] and titles, which have been used as a technique in other reviews to find relevant literature that focus on a specific topic [24–26]. We focused our review on adults with decision-making capacity receiving medical care that aims to maintain or restore health by treatment or prevention of disease [27]. We therefore excluded articles that have target populations under the age of 18 and/or focused on non-medical care (e.g., treatment of women in shelters) or dental care. Empirical (qualitative and quantitative) studies, reviews, policy reports, and evidence-based commentaries were included. Non-research based articles and discussions were excluded [28]. Two reviewers separately searched for articles and excluded articles based on eligibility criteria to ensure a systematic and replicable literature retrieval process.

### Data analysis

We used inductive content analysis to extract, analyze, and interpret data from the articles that met inclusion criteria. Content analysis is a systematic research method that allows researchers to make valid inferences from data by translating context-specific information into general categories that can be combined into a general statement [29]. This method has three phases: preparation, organizing, and reporting. In the preparation phase, articles were chosen as the unit of analysis and read through to obtain a sense of the data. Article characteristics were extracted, including purpose, methodology, country, healthcare setting, care description, and target population. Next, data on how articles conceptualized appropriate care was organized through coding, category creation and abstraction. After thorough examination of article content, articles’ conceptualization of appropriate care was recorded through a summary definition. From this content, themes and subthemes were further abstracted into main categories. Major themes were inductively constructed from the emerging categories. Abstraction was performed by JR-P and repeated by TJ for a subsample to validate method reliability.

## Results

### Literature retrieval

**Fig. 1:**
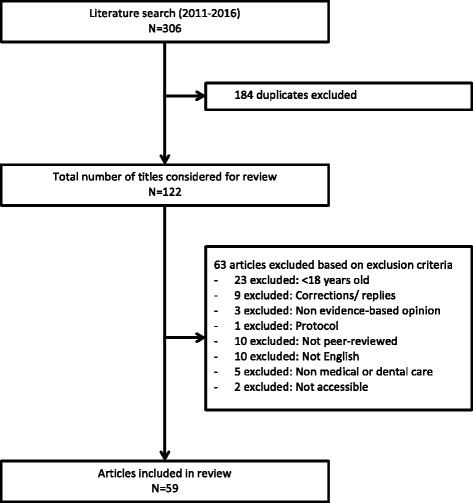
Flow diagram of literature selection

The literature search yielded 306 articles published between since 2011 and 2016 (Fig. 1). After filtering for duplicate records, 122 articles were considered for review. Sixty-three of these articles were subsequently excluded because they did not meet eligibility criteria. Fifty-nine articles were eligible for abstraction [5, 30–87].

### Study description

The reviewed literature included quantitative studies (*N* = 21), qualitative studies (*N* = 6), mixed methods designs (*N* = 7), case studies (*N* = 2), reviews and policy papers (*N* = 14), and commentaries (*N* = 9) (Table 1). Thirty of the articles were either conducted in the US (for empirical studies) or written from the US perspective, and 18 were conducted in or based on a perspective from other industrialized countries, including Canada (*N* = 6), Australia (*N* = 6), Italy (*N* = 5), England or the UK (*N* = 5), Japan (*N* = 2), Israel (*N* = 2), the Netherlands (*N* = 1), Switzerland (*N* = 2), Germany (*N* = 1), Ireland (*N* = 1) and the European region (*N* = 2) (Table 1). Five articles spanned more than one country or had an international focus and only one study took place in a developing nation, (i.e., Afghanistan).

**Table 1:** Description of articles

Author, year	Type of article	Purpose	Country	Setting	Perspective	Type of care	Target population
Ackermann, 2012 [46]	Commentary	To challenge the use of mere clinical practice guidelines to inform quality measurement and performance assessment in primary care	Australia	Primary	System, clinical	Not specified	GP patients
Anstey et al., 2015 [51]	Cross-sectional study using surveys	To determine the extent and characteristics of perceived inappropriate treatment among ICU doctors and nurses	USA	Inpatient	Clinical	ICU care	ICU patients
Barber et al., 2015 [52]	Mixed method, qualitative, review	To develop key performance indicators to evaluate centralized intake systems for patients with osteoarthritis and rheumatoid arthritis	Canada	Various	System	Various	Patients with osteoarthritis and rheumatoid arthritis
Bateson, 2013 [39]	Review	To evaluate the potential role of GPs to reduce unnecessary female genital surgery, while also providing culturally sensitive care	Australia	Primary	Clinical	Procedure counseling and referral	Women who have had or are requesting genital cutting
Bonvicini et al., 2014 [30]	Observational, population-based study	To compare Caesarian section (CS) and ultrasound scan utilization in a public vs. private model of care and the association of use with perinatal outcomes	Italy	Specialized, inpatient, integrated	Clinical	Use of ultrasound and frequency of CS during prenatal care and delivery	Women giving birth in Reggio Emilia Province
Bradford et al., 2015 [53]	Qualitative methods and consensus process	To customize the existing IT-enabled cardiac rehabilitation program delivered by mobile phone through a smartphone app to make it culturally relevant and suitable for Indigenous Australians living in urban and remote communities	Australia	Rehabilitation, Remote care	Patient	Remote cardiac rehabilitation	Indigenous Australians
Breivik et al., 2013 [40]	Review	To make a case for prioritizing chronic pain management in Europe, outline strategies to overcome barriers to effective pain care, and address the confusion of proper uses of opioid medications	Europe	Primary, specialized, inpatient, integrated, other	System	Chronic pain management therapies	European adults with chronic pain
Brien et al., 2014 [41]	Review	To conduct a scoping review to map Canadian research and related activity on system-level appropriateness of care	Canada	Not specified	System	Not specified	Patients in Quebec
Brindis et al., 2011 [42]	Review	To evaluate how Maryland hospitals dealt with issues of inappropriate use of cardiac procedures through new policy initiatives	USA	Inpatient	System	Percutaneous coronary intervention (PCI) and stenting	Patients that have received or may prospectively need PCI
Broekhuis et al., 2014 [54]	Cross-sectional study using surveys	To study the appropriateness of walk-in clinic visits in Quebec, Canada	Canada	Walk-in clinics	Clinical; patient	General	Not specified
Brooks et al., 2013 [73]	Qualitative, mixed methods	To provide a model for adapting remote monitoring to specific populations who are undergoing care for post-traumatic stress disorder (PTSD)	USA	Telehealth	Clinical; patient	Telehealth monitoring for PTSD	American Indian Veterans with PTSD
Chen, 2011 [43]	Review	To evaluate the medical, functional, and quality of life costs of Parkinson's Disease and to discuss treatments that help manage better outcomes	USA	Primary, specialized, integrated, other	System; clinical	Parkinson's disease management therapies	Adults with Parkinson's disease
D’Alleyrand & O’Toole, 2013 [44]	Review	To evaluate the treatment trends in the literature on appropriate timing of (femoral) fractures in polytrauma patients following an injury and discuss the new concept of Early Appropriate Care	USA	Inpatient	Clinical	Fixation surgery for femoral fractures	Polytrauma patients with femoral fractures
Fanari et al., 2015 [76]	Case studies	To highlight how quality measures that aim to decrease Door-to-Balloon-Time (the time from suspected myocardial infarction presentation to primary coronary intervention) may result in poor outcomes due to rushed triage decisions	USA	Inpatient	Clinical	Procedures in the coronary catheterization lab	Patients with suspected myocardial infarction
Fuchs, 2011 [47]	Commentary	To discuss how doctors can be incentivized to provide appropriate care utilization given the dilemma of fulfilling the commitment to the primacy of patient welfare and providing cost-effective care	USA	Not specified	System; clinical	Not specified	Not specified
Hosaka et al., 2011 [31]	Retrospective data analysis	To evaluate the association between the number of blood cultures collected and the appropriateness of care for suspected bacteremic community-acquired urinary tract infection (UTI) in the elderly	Japan	Inpatient	Clinical	Blood cultures collection and UTI treatment	Elderly patients with suspected UTI
Hubbard & Jatoi, 2012	Commentary	To discuss why adjunctive chemotherapy is less used in the elderly than in younger populations	USA	Specialized	Clinical	Adjunctive chemotherapy for colon cancer	Older (vs. younger) colon cancer patients
Kazandijian & Lipitz-Snyderman, 2011 [55]	Review	To discuss the usefulness of health care information technology in assisting care providers to minimize uncertainty while simultaneously increasing efficiency of the care provided	USA	Inpatient	System; clinical	Not specified/ general	Inpatient
King et al., 2013 [32]	Pre-post test	To institute and assess the impact of a process improvement project for blood utilization to ensure appropriateness in transfusion practice	USA	Inpatient	Clinical	Blood transfusions and red blood cell unit usage	Anemic patients, patients that may need transfusions
Korst et al., 2015 [56]	Cross-sectional survey	To examine the extent to which hospitals could be classified by increasingly sophisticated maternal levels of care	USA	Inpatient	Clinical	Perinatal care	Women giving birth in California hospitals
Korst et al., 2015 [57]	Conceptual framework and quantitative survey	To describe the resources and activities associated with childbirth services	USA	Inpatient	Clinical	Perinatal care	Women giving birth in California hospitals
Liang et al., 2012 [33]	Longitudinal study	To examine the racial/ ethnic differences in prostate-specific antigen (PSA) testing and follow-up in primary care practices serving an indigent population	USA	Primary	System; clinical	PSA testing and follow-up	Indigent men in South Texas
Lin & Harris, 2015 [58]	Commentary	To address the issues of variation in interpretation when applying appropriate use criteria in cardiology diagnostic imaging	USA	Not specified	System; clinical	Cardiology diagnostic imaging	Cardiology patients
Lippi & Favaloro, 2011 [49]	Commentary	To identify problems associated with diagnosing bleeding disorders and suggest possible solutions	Italy, Australia	Primary, specialized, integrated, other	Clinical	Diagnosis of bleeding disorders	Patients with hemophilia
Martin, 2012 [59]	Qualitative study using in-depth interviews	To explore older Iranian immigrants' perceptions/ experiences of discrimination in their encounter with the American health care system	USA	Not specified	Patient	Not specified/ general	Iranian immigrant patients that immigrated after age 50, All had health insurance
Mancuso et al., 2016 [71]	Quantitative	To investigate the relationship between care appropriateness and productivity evolution in public hospital services in 20 Italian region systems for the period 2008-2012	Italy	Inpatient	System	Not specified	Not specified
Mataoui & Sheldon, 2016 [60]	Commentary	To call attention to the importance of oncology nurses to develop a deeper understanding of the cultural practices and health beliefs of Muslim patients when providing cancer care	USA	Not specified	Patient	Oncology/ cancer care	Muslim cancer patients
Matthie, 2015 [78]	Literature review and case studies	To highlight prominent issues of pain treatment for sickle cell disease (SCD) and make recommendations to hospital nursing staff on how to improve care for adults with SCD	USA	Inpatient	Clinical; patient	Sickle cell disease pain treatment	Patients with presenting with sickle cell disease related pain episodes
McCormick, 2014 [61]	Commentary	To call attention to the importance of culturally sensitive care and identify tips for cultural sensitivity	USA	Not specified	Patient	Not specified/ general	Culturally diverse, older patients
McFadden et al. 2013 [62]	Qualitative study using interviews	To explore the extent to which cultural context makes a difference to experiences of breast-feeding support for women of Bangladeshi origin and to consider the implications for the provision of culturally appropriate care	England	Inpatient, community/ home-based care	Patient	Maternity care and breast feeding support	Breast feeding women of Bangladeshi origin
Mitchell et al., 2016 [79]	Quantitative	To evaluate the diagnostic outcomes and therapeutic decisions made after a repeat pancreatic cancer testing using endoscopic ultrasound-guided fine-needle aspiration (EUS-FNA) for patients that have undergone a prior testing with inconclusive results	Canada	Inpatient	Clinical	Endoscopic ultrasound-guided fine-needle aspiration (EUS-FNA)	Patients undergoing EUS-FNA after initial testing for pancreatic cancer was inconclusive
Mochizuki, 2012 [63]	Review	To describe current cultural issues in Japanese health care services that have resulted from increased immigration	Japan	Not specified	System; patient	Not specified	Foreigners, ethnically, culturally diverse patients
Morgan et al., 2015 [64]	Retrospective database cross-sectional analysis	To establish the prevalence and nature of pathology test-ordering of GP trainees, and to describe the associations of this test-ordering (in the context of increasing over-testing and implications for patient safety)	Australia	Primary (urban and rural settings)	Clinical	Not specified/ general	Not specified
Nahm et al., 2011 [34]	Retrospective database cross-sectional analysis	To examine the effects of timing of fixation and investigate risk factors for complications	USA	Inpatient	Clinical	Femur fracture stabilization in patients with multiple injuries	Patients with femoral fractures
Newbrander et al., 2014 [65]	Qualitative study	To explore traditional practices of women, families, and communities related to maternal and newborn care, and sociocultural and health system issues that create access barriers	Afghanistan	Home versus health care facilities	System; patient	Perinatal, antenatal, and newborn care	Women giving birth or have recently given birth, newborns
Panella et al., 2012 [35]	Multi-center cluster-randomized trial	To evaluate whether Clinical Pathways improve the outcomes and the quality of care provided to patients after acute ischemic stroke	Italy	Inpatient	Clinical	Post-acute ischemic stroke care	Patients that have just had an acute ischemic stroke
Pape et al., 2016 [80]	Commentary	To critique the parameters of the Early Appropriate Care protocol for determining whether patients are cleared for stabilization surgery	Germany	Inpatient	Clinical	Surgical stabilization of fractures	Trauma patients with fractures
Paprica et al., 2015 [66]	Literature review, consensus process	To explore whether the direct involvement of policy stakeholders could advance appropriateness and disinvestment	Canada	Not specified	System	Not specified	Patients in Canada
Piers et al., 2011 [5]	Cross-sectional survey	To determine the prevalence of perceived inappropriateness of care among intensive care unit (ICU) clinicians	Europe, Israel	Inpatient	Clinical	ICU services	ICU patients
Pittet et al., 2015 [87]	Quantitative questionnaire of a simulated case and qualitative methods using focus groups	To explore how treatment decisions of practicing gastroenterologists differ from those of experts, using a vignette case study and a focus group	Switzerland	Specialized	Clinical	Gastroenterology; treatment of Crohn's disease and ulcerative colitis	Hypothetical Crohn's disease and ulcerative colitis patients
Poulos et al., 2011 [36]	Cohort study	To report utilization review data on inpatients in acute care with stroke, hip fracture or elective joint replacement, and other inpatients referred for rehabilitation	Australia	Inpatient, integrated, other	Clinical	Readiness of transfer to rehabilitation	Inpatients in acute care with stroke, hip fracture or elective joint replacement
Reich et al., 2016 [81]	Cohort study	To evaluate whether Early Appropriate Care protocol for stabilizing fractures in patients with advanced age require unique parameters to mitigate complications	USA	Inpatient	Clinical	Surgical stabilization of fractures	Skeletally mature trauma patients with unstable fractures
Russo et al., 2016	Quantitative survey	To investigate the interplay between perceptions of individual employees regarding HR practices and the variability of such perceptions within the department and their effects on appropriateness of care	Italy	Inpatient	Clinical	Not specified	Not specified
Sandela et al., 2012	Cross-sectional analysis of simulated case	To investigate the appropriateness and cost of care and quantify their relationship to performance based on a simulated case	USA	Primary	Clinical	Simulated case of a 45-year-old man complaining of right-sided localized chest pain	Simulated case
Schneider, 2014 [83]	Case studies	To illustrate the importance of early digestive tract assessment impact on the outcomes of liver transplantation after acetaminophen poisoning	UK	Inpatient	Clinical	Emergency liver transplantation after acetaminophen poisoning	Patients needing liver transplants who suffer acetaminophen poisoning
Schoormans et al., 2013 [50]	Commentary	To discuss care provision problems of congenital heart disease (CHD) patients lost to follow-up, those receiving too little care, and those receiving too much care, and offers appropriate and cost-effective health care delivery targets	Netherlands	Primary, specialized, inpatient, integrated, other	System; patient	CHD long-term treatment and management	Adults with CHD
Sharpe & Uchendu, 2014 [45]	Review	To address the issues of discrimination and inadequate health care provision for LGBT veterans through new policies that align with the Veteran’s Health Administration's Strategic Plan 2013-2018	USA	Not specified	System; patient	Not specified	LGBT Veterans
Tasker et al., 2014 [67]	Review	To review evidence of performing damage control orthopedics versus definitive stabilization and the use of Early Appropriate Care protocols	UK	Inpatient	Clinical	Stabilization of fractures	Polytrauma patients with fractures
Tolson et al., 2011 [68]	Policy paper	To report the outcomes of a workshop by the International Association of Gerontology and Geriatrics about recommendations for improving quality of care experiences for older people in nursing homes around the world	International	Nursing homes	System; patient	Geriatric care, pain management, end of life care	Residents of nursing homes
Trinh et al., 2014 [74]	Quantitative survey and qualitative interviews	To describe the challenges implementing the Culturally Focused Psychiatric Consultation Program for depressed Latino and Asian Americans in four urban primary care practices	USA	Primary	Patient	Psychiatric consultation for depression	Latino and Asian Americans
Trinh et al., 2015 [75]	Qualitative interviews	To evaluate participant acceptability of a Culturally Focused Psychiatric Consultation Program for depressed Latino Americans	USA	Primary	Clinical; patient	Psychiatric consultation for depression	Latino Americans
Tucker et al., 2013 [69]	Review	To report the literature review findings of examples of the balance of care approach framework during a 40-year time span	Mostly UK, Ireland, Canada	Various	System	Various	Various (health, social, and mental care)
Vallier et al., 2013 [38]	Statistical modeling based on retrospective database cross-sectional analysis	To define which clinical conditions warrant delay of definitive fixation for pelvis, femur, acetabulum, and spine fractures and develop a model to predict complications	USA	Inpatient	Clinical	Definitive fixation for pelvis, femur, acetabulum, and spine fractures	Adults with pelvis, acetabulum, spine, and/or proximal or diaphyseal femur fractures
Vallier et al., 2015 [70]	Prospective study	To review initial experiences with a protocol (to determine the timing of definitive fracture care based on the adequacy of resuscitation) with adherence to the protocol and assess barriers to implementation	USA	Inpatient	Clinical	Definitive fixation of pelvis, acetabulum, spine and femur fractures within 36 hours of injury	Polytrauma, adult patients with fractures
Vallier et al., 2016 [84]	Prospective study	To evaluate whether a standardized protocol for fracture care would enhance revenue by reducing complications and length of stay	USA	Inpatient	Clinical	Surgical stabilization of fractures	Trauma patients with femur, pelvis or spine fractures
Vaucher et al., 2016 [71]	Qualitative study using focus groups	To explore and compare gastroenterologists’ and patients' perceptions of risks and benefits of treatments and prioritizations of expected outcomes	Switzerland	Specialized	Clinical; patient	Treatment of inflammatory bowel disease including ulcerative colitis and Crohn's disease	Patients with ulcerative colitis and Crohn's disease
Weideman et al., 2015	Mixed methods	To design, implement, and evaluate a virtual simulation experience facilitating student access to diverse cultures and strengthening their ability to provide culturally congruent care.	USA	Specialized; Virtual simulation of pre- and post-natal care	Clinical; patient	Pre- and post-natal care	Simulated Amish and African American patients
Weinberg et al., 2015 [72]	Case control study	To better characterize the relationship between post-operative complications and the time required for resuscitation of metabolic acidosis using the Early Appropriate Care protocol	USA	Inpatient	Clinical	Treatment of orthopedic fractures	Trauma patients with orthopedic fractures
Wynell-Mayow, et al., 2016 [85]	Pre-post test	To assess the impact of the Cambridge Polytrauma Pathway on quality process indicators	UK	Inpatient	Clinical	Treatment of orthopedic polytrauma	Trauma patients with orthopedic fractures

### Article characteristics

Table 1 provides a description of the articles included in the review. Of the articles that specified health care setting, most took place in the hospital (*N* = 30). Other settings included primary care (*N* = 11), secondary or specialized care (*N* = 8), integrated care or care that took place in more than one setting (*N* = 8), other types of care settings such as home health, nursing homes, urgent care walk-in clinics, and remote care (i.e. telehealth) (*N* = 13), and settings that were not specified (*N* = 12). Articles focused on therapeutic procedures (e.g., stenting, fracture stabilization surgery), diagnostic testing (e.g., PSA testing for prostate cancer, blood culture collection for UTI diagnosis), condition management or monitoring (e.g., chronic pain management, telehealth monitoring for PTSD), setting - specific care (e.g., intensive care unit services and primary care services), and age-specific care (e.g., geriatric care). Most articles related to specific health conditions (*N* = 38), including orthopedic fractures (*N* = 11), obstetrics and maternity care (*N* = 6), cardiac and cardiovascular conditions (*N* = 7), cancer (*N* = 4), mental health (*N* = 3), pain management (*N* = 2), bleeding disorders (*N* = 2), gastrointestinal disorders (*N* = 2), and other medical conditions (i.e., sickle cell disease, Parkinson’s disease, arthritis, liver failure, and urinary tract infection). Eleven of the 59 articles focused on minority patients or populations, six articles targeted older patients, four articles focused on women and one focused on men. Most articles defined appropriate care from a clinical perspective (*N* = 39), more than a third of studies defined appropriate care from the health system perspective (*N* = 22), and slightly less than a third were defined from the patient perspective (*N* = 16). Sixteen articles represented more than one perspective.

### Main results

**Fig. 2:**
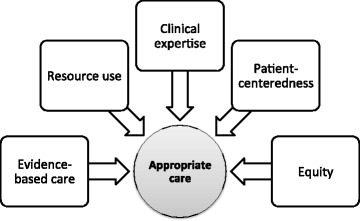
Categories of appropriate care

During the review process, five categories emerged from the inductive content analysis of the articles’ full text. These categories included evidence-based care, patient-centeredness, clinical expertise, effective use of resources, and equity (Fig. 2).

#### Evidence-based care

**Fig. 3:**
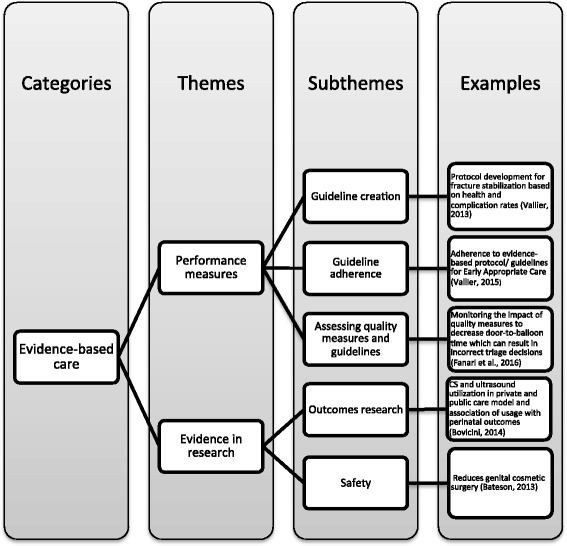
Evidence-based care

Forty articles discussed elements of evidence-based care, which we define as care that is proven to improve health outcomes. Evidence-based care included outcomes research, the assessment and use of evidence-based standards (i.e., guidelines, quality indicators), and the use of scientific evidence in treatment (Fig. 3). Appropriate care was determined based on positive health outcomes, adherence to evidence-based guidelines, and applying evidence in practice. One prominent theme in the outcomes-based literature was creating and testing Early Appropriate Care, an evidence-based protocol for timing stabilization of fractures after traumatic injury. Other studies focused on evaluating clinical effectiveness and guideline adherence from a systems-level perspective to reduce unnecessary care [33, 55, 69, 79] and decrease outcome variation [35, 36, 40, 49, 53, 55, 86] and from a clinical perspective to ensure safe and effective care [34–36, 41, 42, 46, 49, 67, 68, 70, 80, 81, 85]. In addition, many articles indicated the need for more evidence and guidelines to guide clinical decisions, especially for populations that are underrepresented in research, such LGBT patients [45] and the elderly [48, 68].

Some commentaries and case studies questioned the ability of evidence and guidelines to account for context or real world disease complexity. For example, commentaries by Lin (2015) and Lippi & Favaloro (2012) discuss guideline interpretation and implementation challenges that can lead to negative outcomes [49, 58] and case studies by Schneider (2014) and Fanari (2015) illustrate how gaps in guidelines can cause clinicians to overlook vital elements of appropriate care, which can lead to poor outcomes if practices are not effectively monitored [76, 83]. Ackerman (2012) also challenged the use of guidelines, stating that guidelines must be combined with clinical expertise and patient values to ensure appropriate care delivery [46].

#### Clinical expertise

**Fig. 4:**
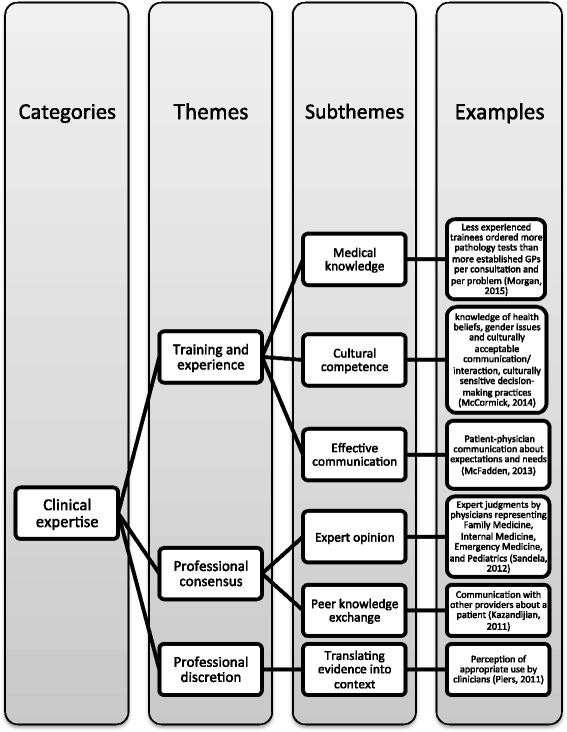
Clinical expertise

Thirty-eight articles discussed the importance of clinical expertise in appropriate care delivery. Articles defined appropriate care in terms of adequate education and training for health care professionals, the use of expert opinion/ professional consensus to guide clinical decisions, and clinician discretion to tailor treatment to patient cases and to manage uncertainty (Fig. 4). Articles emphasized the importance of education and training in specialty medical fields [68], the proper use of guidelines and protocols [32], and cultural competence and effective communication to help clinicians identify patient-specific risks and needs, align treatment goals, and enable shared decision-making [39, 45, 55, 59–63, 68, 71, 74, 75, 78, 86]. To ensure effective communication within the therapeutic relationship, articles also discussed the need to overcome language barriers [62, 63, 74, 75].

Professional discretion was viewed as an important element of appropriate care that enables clinicians to assess necessity [5, 36, 47, 51, 54, 66], translate evidence for specific patient risks, needs, and goals [36, 43, 45, 46, 55, 66, 68, 71, 87], balance patient needs with costs [47], and manage uncertainty [30, 43, 47, 55, 64, 66].

Professional consensus and knowledge exchange appeared throughout the literature as tools for making appropriate care decisions to reduce variation in service use [41, 42, 58, 66], confirm indications [37, 64], coordinate care [73], manage uncertainty [43, 55, 64], and create standards and guidelines [33, 34, 36, 46, 53, 84].

#### Patient-centeredness

**Fig. 5:**
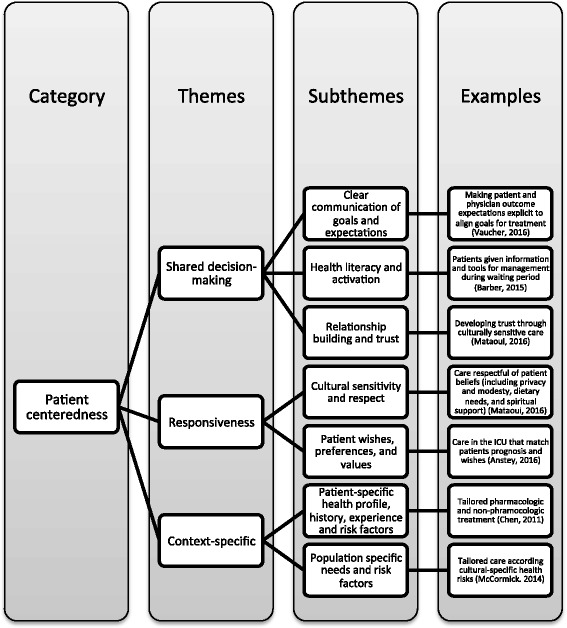
Patient-centeredness

Considerations of patient-centered care were present in about half of the reviewed articles (*N* = 30). Elements of patient-centeredness included providing patients with context-specific, responsive, coordinated care and supporting patient autonomy through open communication and shared decision-making (Fig. 5). Context-specific care tailors health care services to patients’ health profile, medical history, and risk factors [33, 36, 43, 45, 49, 55, 61, 62, 64, 68, 87]. Responsiveness refers to culturally sensitive and respectful care that accounts for patient values, culture, needs and preferences. Responsiveness was especially emphasized in articles that focused on providing culturally appropriate care to various groups, including immigrant minorities [59, 61–63], LGBT veterans [45], and women in Afghanistan [65]. Coordinated and integrated care involves managing health and social services across conditions and settings [36, 39, 40, 43, 50, 68, 73] (Fig. 5). Other elements of patient-centered care included shared decision-making through open communication of goals and expectations [49, 55, 60, 68, 71] that help identify patient perceptions and acceptability of care [40, 43, 50, 53, 54, 60, 61, 68, 71], health literacy and patient activation [33, 52, 65], and building a relationship of trust with providers [45, 60, 61, 65]. Patient-centered care requires patient empowerment and engagement through disease prevention and self-management tools, education, and effective communication.

#### Resource use

**Fig. 6:**
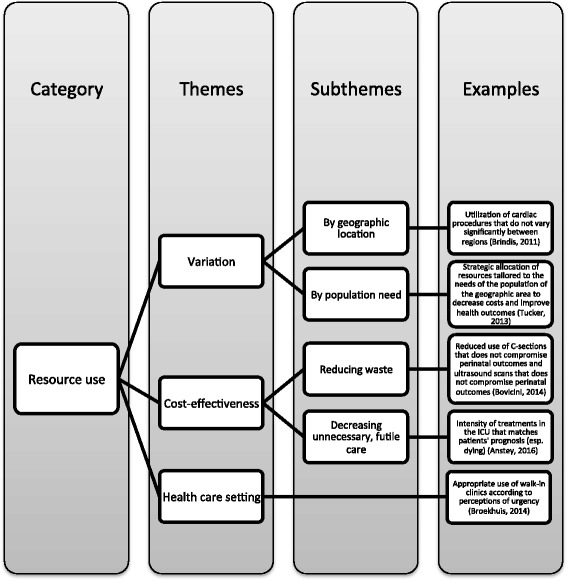
Resource use

The role of resource use in determining appropriate care was discussed in 33 articles. Subthemes included variation in resource use, cost-effectiveness, and health care setting (Fig. 6). Twenty articles discussed variation in resource use to reduce waste and unnecessary care and ensure proper provision [33, 40, 42, 47, 50, 56–58, 64, 66, 82] and to assess equity in health care delivery practices [33, 45, 50, 58, 63]. Cost-effectiveness was discussed in terms of allocating resources at the health system level [41, 66, 69, 77], making clinical decisions in practice [37, 43, 47, 58, 82], and decreasing cost in damage care orthopedics [67].

#### Equity

**Fig. 7:**
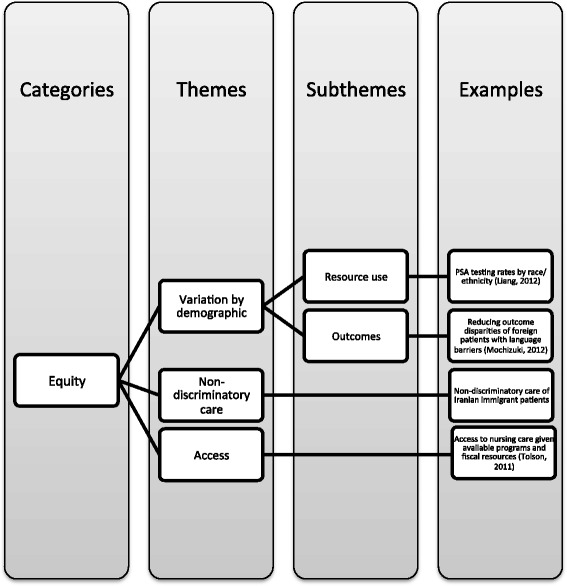
Equity

Equity was discussed in 14 articles. This category included many themes that overlap previously discussed themes, including demographic and geographic variation in resource use [33, 40, 42, 50, 58, 63] and health related outcomes [33, 45, 50, 57, 60, 63], access to health care services [33, 45, 52, 60, 63, 65, 68, 75, 74, 78, 79], and non-discriminatory care [45, 59, 62, 78] (Fig. 7).

## Discussion

Using content analysis, this integrative review identified emerging themes from the literature to inform a more integrated approach to appropriate care. Although the use of appropriate care in the literature varied, our review revealed five emerging categories: evidence-based care, clinical expertise, patient-centeredness, resource use, and equity, which were employed in varying combination with overlapping themes and subthemes (Figs. 3, 4, 5, 6 and 7). These elements correspond with the IOM’s performance targets of proving safe, effective, patient-centered, timely, efficient, equitable care and provides guidance for how systems can achieve the IHI’s Triple Aim of improving population health, improving experiences of care, and decreasing per capita costs [7, 8].

Most articles conceptualized appropriate care from a clinical perspective using outcomes research, peer consensus, and guideline adherence to determine whether care was appropriate. The system perspective defined appropriate care in terms of guideline adherence, cost-effectiveness, and reduced variation in resource use and outcomes between geographic regions, health care facilities, and demographic groups. These findings contrast with findings from the review by Sanmartin et al. (2008) that found appropriateness of care to be most often defined according to RAND/UCLA Appropriateness Methods [21], and better correspond with Brien et al.’s (2014) review of system level appropriateness in Canada, which found appropriate care to be defined in terms of health services utilization, accordance with guidelines, and cost-effectiveness [41]. However, unlike past reviews, this review found more representation of the patient perspective that focused on tailoring evidence-based care to account for patient needs and preferences and providing culturally sensitive care.

Emphasis on research outcomes and evidence-based guidelines conveyed a reliance on evidence-based measures to mitigate uncertainty in clinical decision-making and reduce variations in health care delivery practices. Researchers with the Dartmouth Atlas Project that investigates variation in care refer to clear-cut evidence - based treatment as effective care and assert that it should always be used in indicated circumstances [88]. However, the review also questioned the sole reliance on evidence to determine appropriateness as insufficient and sometimes even dangerous. Limited evidence for certain populations and conditions, as well as disease - specific guidelines were shown to not always account for disease complexity and patient variability and leave a degree of ambiguity and uncertainty that must be qualified by clinician discretion, patient input, and effective monitoring. Research by other authors also discusses the limits of evidence for providing patient-centered care. Reeve and colleagues (2013) found that English primary care physicians are skeptical of “tick-box” models of care that evaluate performance based on disease-specific guidelines, because they are often unable to account for the high degree of complexity and uncertainty that is common in primary care [89]. Reeve (2010) therefore espouses the use of Interpretive Medicine that allows physicians to use a range of evidence and context-specific knowledge to interpret patients’ experience of illness [90].

Furthermore, findings from our review suggest that patient input and expertise may be able to guide appropriate care decisions. Articles in the review discussed the role of patients in determining appropriate care when different options with varying long and short-term effects exist, such as therapies for Parkinson’s Disease [43], chronic gastrointestinal conditions [71], or end of life care [68]. Anstey et al. (2016) and Piers et al. (2011) found that effective communication with patients’ families about end of life care could also decrease overuse of unnecessary or futile care in the intensive care unit [51, 5]. In the US context, the Dartmouth Atlas developed the term preference sensitive care to describe care with many viable options and tradeoffs that can only be deemed appropriate by the patient [91]. Preference sensitive care not only ensures that care is appropriate for patient - specific needs and goals, but also helps to curb unnecessary variation in services due to resource availability and perverse incentives for providing care. Empowering patients to take an active role in health care seeking and decisions can also contribute to appropriate care delivery by providing patients with education and tools to overcome barriers to access (e.g., Afghani women requiring perinatal services [65]); manage chronic conditions (e.g. people with arthritis waiting to receive services [52]) ; understand risks of elective procedures (e.g. women seeking genital surgery [39]); and communicate their health needs and risk factors without fear of discrimination (e.g., LGBT veterans [45]). Furthermore, the Choosing Wisely campaign has tried to harness patient expertise to mitigate overuse by providing patients with a list of relevant questions to ask their doctors when they are making specific health care decisions [17].

Although evidence-based care, professional expertise, patient-centeredness, resource use, and equity were discussed across health care contexts, conceptualizations of how these elements should be applied varied by health care system especially in terms of appropriate allocation of resources, reinforcing Sharpe’s (1997) claim that system level appropriateness is shaped by system values and priorities for resource allocation and equity [22]. Appropriate care in health systems with tax-based financing (e.g. Italy, Australia, Canada, England) emphasized monitoring cost-effectiveness, while appropriate care in more market-based health systems (e.g. USA, the Netherlands) focused on reducing resource and outcome variation. Furthermore, the use of provider incentives was discussed in the light of the relative country context. Fuchs (2011) advocated for the use of capitation in the US to curb costs and replace traditional fee-for-service models [47]. However, because managed care has come under scrutiny in the US for cutting costs at the expense of quality, the Affordable Care Act has launched new models of delivery that tie quality to remuneration and provide opportunities for providers to share savings [90]. Conversely, Ackermann (2012) discussed how performance-based incentives in the Australian context could facilitate unintended “perverse” incentives to over-treat or undertreat, giving the example of how the Medicare Benefits Schedule remunerates practitioners for Type 2 Diabetes screening if the screen is positive, creating an incentive not to screen and to overdiagnose [46]. Pape et al. (2016) illustrated how even clear-cut evidence-based guidelines, such as Early Appropriate Care for determining the timing of fracture surgery, can be context - specific due to the use of different emergency room procedures in different countries [80]. Furthermore, countries with large minority communities (i.e., USA, Australia, England) or rising rates of immigration (i.e., USA, Japan) emphasized the importance of cultural competence and respect for delivering appropriate care to diverse patients [53, 59–63, 73, 45].

Although understandings of appropriateness inevitably vary by context, the review gleaned implications for appropriate care provision. The importance of evidence-based care and guidelines to support clinical decision-making points to a need for further investment in research and infrastructure that make evidence accessible to health care practitioners. Guideline and protocol development should also include clinician input on implementation challenges, education and training, and feedback mechanisms [32] to prevent against misuse and misinterpretation that can lead to inappropriate diagnosis and care [58]. In addition, increased awareness of patient diversity and unique needs require medical schools and continuing education programs to include cultural competency and communication training to facilitate person-based care and shared decision-making.

This review considers the insights from varying perspectives of appropriate care to create a more comprehensive view of appropriate care delivery that includes every level of the health care system. However, this review is limited by its focus on adult populations, English language literature, specific search terms, and publication years. Future research could employ more scoping review methods to evaluate the use and understanding of appropriate care and how it changes according to population and context.

## Conclusion

Although conceptualizations of appropriate care vary in the literature, they are often characterized by evidence-based care, clinical expertise, patient-centeredness, resource use, and equity. Evidence-based care is essential to providing appropriate care, but must be qualified by clinician discretion, respect for patient wishes and values, and context - specific concepts of equitable distribution of resources. This integrated understanding of appropriate care can help inform policy and clinical delivery practices according to context-specific means and priorities.

## Abbreviations

CHD: Congenital heart disease
CP: Clinical pathways
CS: Caesarian section
GP: General practitioner
ICU: Intensive care unit
LGBT: Lesbian, gay, bisexual, and transgender
LOS: Length of stay
PCI: Percutaneous coronary intervention
PSA: Prostate-specific antigen
QOL: Quality of life
UTI: Urinary tract infection
VHA: Veterans health administration
